# Supplementary material for: Quantifying Functional Impairment of ABCA3 Variants Associated with Interstitial Lung Disease
Source: Int J Mol Sci. 2023 Apr 20;24(8):7554. doi: 10.3390/ijms24087554 (PMC10141231; doi:10.3390/ijms24087554)
Supplement: Supplementary file 1 [file ijms-24-07554-s001.zip › ijms-2344198-supplementary.pdf]

SUPPLEMENT

Table S1. Assays to characterize ABCA3 intracellular trafficking and pumping function

Code	Name of assay	Method	Read out	Biochemical meaning
A	% ER localization of ABCA3	Co-staining immunofluorescent ABCA3^{+} vesicles and ER markers calnexin [1, 2]	Percentage of ABCA3^{+} vesicles colocalized with calnexin in relation to all ABCA3^{+} vesicles	Percentage of ABCA3 localized within ER [1, 2]
B	N-glycosylation of ABCA3	N-glycosylation of ABCA3-HA after digestion with endoglycosidase H (EndoH) or N-glycosidase F (PNGaseF) (Western blot) [2, 3]	Ratio of complex oligosaccharide protein (190 kDa) to hybrid oligosaccharide protein (190 kDa + 180 kDa) after EndoH digestion.	Presence of exclusive 180 kDa band after PNGaseF digestion indicates N-glycosylation of ABCA3.<br>Presence of remaining 190 kDa band after EndoH digestion demonstrates presence of complex oligosaccharide, indicating processing (N-glycosylation) in Golgi. [2-7]
C	% lysosomal localization of ABCA3	Co-staining immunofluorescent ABCA3^{+} vesicles and lysosomal marker CD63 in cells (confocal microscopy) [3]	Percentage of ABCA3^{+} vesicles colocalized with CD63 in relation to all ABCA3^{+} vesicles or dots	Percentage of ABCA3 processed to CD63^{+} intracellular vesicle membrane [1-3, 6, 8-11]

D	Proteolytic cleavage of ABCA3	Proteolytic cleavage of ABCA3 protein (Western blot) [2]	Ratio of 170 kDa proteolytically cleaved protein band to 190 kDa non-cleaved protein band	Presence of lower band demonstrates expression of ABCA3, its transport from ER to MVB/LB [1-4, 12-15], and its proteolytic processing, likely after ABCA3 mediated lipid transport and build of LBs.
E	Volume of ABCA3+ vesicles	Volumes of immunofluorescent ABCA3+ vesicles located inside cells (confocal microscopy) [2, 13]	Volume of ABCA3+ vesicles	Normal or abnormal size and structural formation of ABCA3+ lysosomal compartment [1, 2, 4, 11-14, 16]
F	Amount of PC from recycling into ABCA3+ vesicles	ABCA3+ vesicles containing 1:20 TopF-PC (confocal microscopy) [17]	Fluorescence intensity of TopF-PC per ABCA3+ vesicles in all ABCA3+ vesicles	PC (from recycling) transport activity of ABCA3+ vesicles [11, 13, 17-19]
G	Amount of PC from *de novo* synthesis into ABCA3+ vesicles	ABCA3+ vesicles containing 1:125 propargyl-choline (confocal microscopy) [16]	Fluorescence intensity of propargyl-choline per ABCA3+ vesicles in all ABCA3+ vesicles	PC (from *de novo* synthesis) transport activity of ABCA3+ vesicles [16, 18, 19]
H	ATPase activity of ABCA3	Vanadate-induced nucleotide trapping and photoaffinity labelling of ABCA3-GFP with 8-azido-[α-32P] ATP or 8-azido-[α-32P]ADP [3, 5, 7]	Vanadate-induced trapping analyzed by autoradiography<br><br>ATPase activity	ATP hydrolysis activity of ABCA3 protein [1, 3, 5, 7, 10]

		ATPase assay: measured as free phosphate released, compared with WT ABCA3 activity, and normalized to Western blot with anti-GFP antibody [1, 10]		

Table S2. Variants of ABCA3 included in this study.

Variant	Model	Reference	Note
T1114S	HEK293	[5]	
R288K	A549	[10]	
	A549	[2]	
	A549	This paper	
G964D	A549	[2]	
	A549	[8]	
R208W	A549	[2]	
E292V	A549	[1]	
	A549	[10]	
	A549	[2]	
	HEK293	[5]	
	A549	[17]	
	A549	[11]	
	A549	[15]	
	A549	This paper	
G667R	A549	[14]	
N568D	A549	[15]	
	A549	[14]	
	HEK293, A549	[3]	
	A549	[16]	
	HEK293	[7]	
L1580P	A549	[14]	

	HEK293	[3]	
	A549	[16]	
F629L	A549	[14]	
T1114M	A549	[14]	
	HEK293	[5]	
R43L	A549	[12]	Volume of ABCA3+ vesicle was not quantified. It was described that "Expression of R43L, R280C and L101P mutations had a negative effect on vesicle formation and induced a lower number of smaller compact LAMP3+ vesicles", and it looked smaller than wild type at confocal image roughly.<br>PC transportation activity: uptake of C12-NBD-phospholipids PC and PE into ABCA3 vesicles in A549 cells transfected with pUB6/HA-ABCA3 vectors was studied by confocal microscopy.
D253H	A549	[9]	Vesicle volume was roughly estimated from electron microscopy image
G1221S	HEK293, A549	[3]	Volume of ABCA3+ vesicle was not quantified. It looked similar to N568D and L1580P at confocal images, therefore absolute value was calculated as the mean value of N568D and L1580P.
N53Q	HEK293, A549	[6]	Similar to wild type cells
R1474W	A549	[10]	R1474W looked similar to WT, having higher absolute % ATPase activity compared to R288K N-glycosylation was defined as normal, without exact value from the paper.
T1173R	A549	[9]	Vesicle volume was roughly estimated from electron microscopy image
R295C	HEK293	[7]	Vesicle volume was roughly estimated from electron microscopy image
E292K	HEK293	[5]	
E690D	HEK293	[5]	
T1114A	HEK293	[5]	
K1388N	A549	[13]	Defined as mis-trafficked mutation
	A549	[2]	Partially mistrafficked with less proteolytically lower band (170 kDa)

	A549	[17]	Able to form vesicles, but these vesicles were significantly smaller and the lipid amount within them was significantly lower than in the cells expressing ABCA3-WT
	A549	[4]	Defined as functional mutation
S1262G	A549	[1]	
D953H	A549	This paper	
N140H	A549	[1]	
F1077I	A549	This paper	
P248S	A549	This paper	
Q1045R	A549	This paper	
C611R	A549	This paper	
P32S	A549	This paper	
E1364K	A549	This paper	
G1421R	A549	[13]	
	A549	This paper	
A1046E	A549	[13]	
	A549	This paper	
V1399M	A549	[1]	
	A549	This paper	
G1314E	A549	This paper	
F1203del	A549	[1]	
R280C	A549	[12]	Volume of ABCA3^{+} vesicle was not quantified. It was described that "Expression of R43L, R280C and L101P mutations had a negative effect on vesicle formation and induced a lower number of smaller compact LAMP3^{+} vesicles", and it looked smaller than wild type at confocal image roughly.<br>PC transportation activity: uptake of C12-NBD-phospholipids PC and PE into ABCA3 vesicles in A549 cells transfected with pUB6/HA-ABCA3 vectors was studied by confocal microscopy.

N124Q	HEK293, A549	[6]	Immunoblot analysis of cell lysates revealed decreases in levels of ABCA3 protein expression by ~50% in single mutants of N124 and N140 and by as much as 85% when both N residues were mutated<br>Volume of ABCA3^{+} vesicle was not quantified. Absolute value was roughly estimated from confocal images.
N140Q	HEK293, A549	[6]	Volume of ABCA3^{+} vesicle was not quantified. Absolute value was roughly estimated from confocal images.
G202R	A549	This paper	
M760R	A549	[13]	
	A549	[13]	
	A549	This paper	
Q215K	A549	[13]	
	A549	[13]	
	A549	This paper	
L101P	A549	[15]	
	A549	[11]	
	A549	[1]	
	HEK293, A549	[3]	
	A549	[12]	
	A549	[10]	
L982P	HEK293, A549	[3]	
G571R	A549	This paper	
Ins1518fs	HEK293, A549	[3]	

L1553P	HEK293, A549	[3]	
Q1591P	HEK293, A549	[3]	
E690K	HEK293	[5]	ATPase activity （fraction of WT nt） absolute value: 2
E292D	HEK293	[5]	
E690R	HEK293	[5]	

Table S3. Five patients without fitting results from figure 6.

Patient Nr.	Allele 1	Allele 2	Trafficking (average)	Pumping (average)	Function (average)	Function (sum)	Clinical outcome (score)	Clinical outcome	Refence
4	R208W	R43H	1	0.655	0.8825	1.655	5	Alive, lung transplant at 4 months	Wambach, JA, et al. 2014
15	E690K	E690K	1	0.625	0.875	1.625	5	Alive, lung transplant at 5 months	Wambach, JA, et al. 2014
26	K1388N	K1388N	0.815	0.685	0.772	1.500	5	Died at 9 weeks	Wittmann, T., et al. 2016
41	G1421R	Q1045R	0.525	0.618	0.574	1.143	5	Died after 1 month	Own unpublished data
46	F1077I	F1077I	0.911	0.1	0.708	1.011	5	Died at 2 months	Wambach, JA, et al. 2014

Legends for supplemental figures

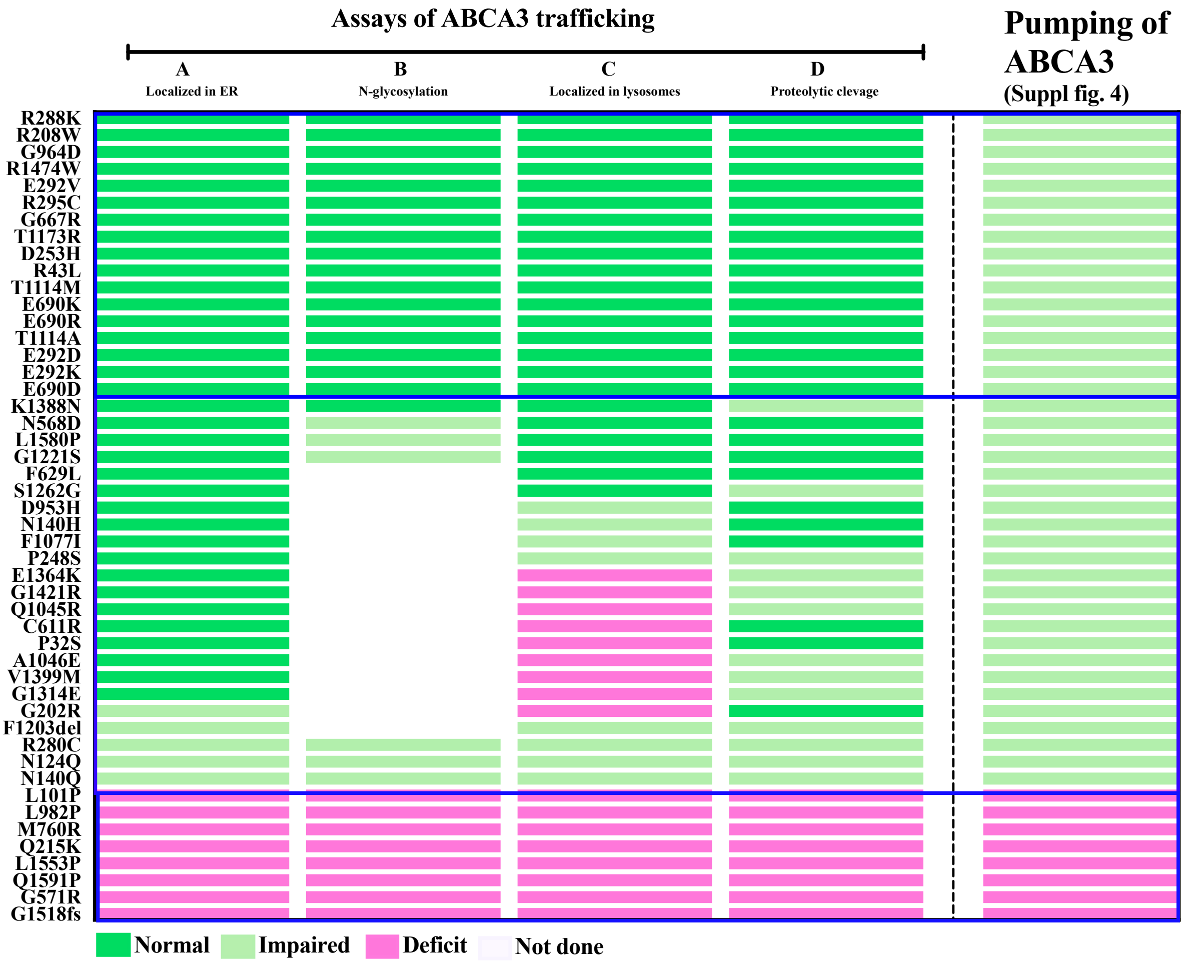

Figure S1: Data of disease related ABCA3 variants with assays on intracellular trafficking and function. All variants had defective ATPase activity. (a) Intracellular trafficking of ABCA3: %localized in ER (A), N-glycosylation (B), %localized in lysosomal compartments (C), proteolytic cleavage (D), and overall function of ABCA3. Dark green square indicated normal (1 $\pm$ 1 nSD), light green square indicated impaired (within 1 $\pm$ 3 nSD), purple square indicated defective (beyond 1 + 3nSD), white square indicated assays not done.

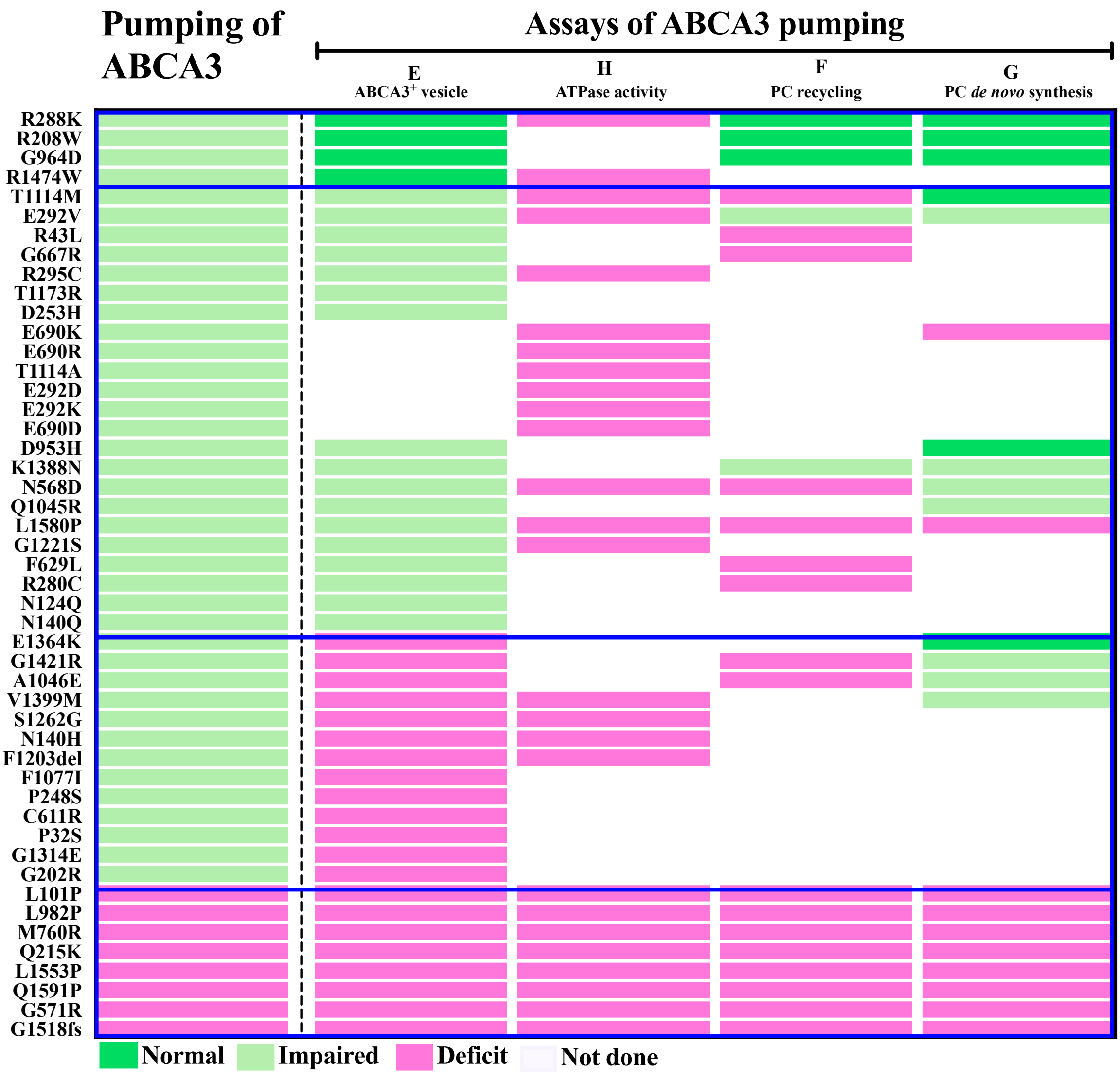

Figure S2: Data of disease related ABCA3 variants with assays on pumping: volume of ABCA3+ vesicles (E), PC from recycling into ABCA3+ vesicles (F), PC from *de novo* synthesis into ABCA3+ vesicles (G), ATPase activity of ABCA3 (H). Dark green square indicated normal (1 ± 1 nSD), light green square indicated impaired (within 1 ± 3 nSD), purple square indicated defective (beyond 1 + 3nSD), white square indicated assays not done.

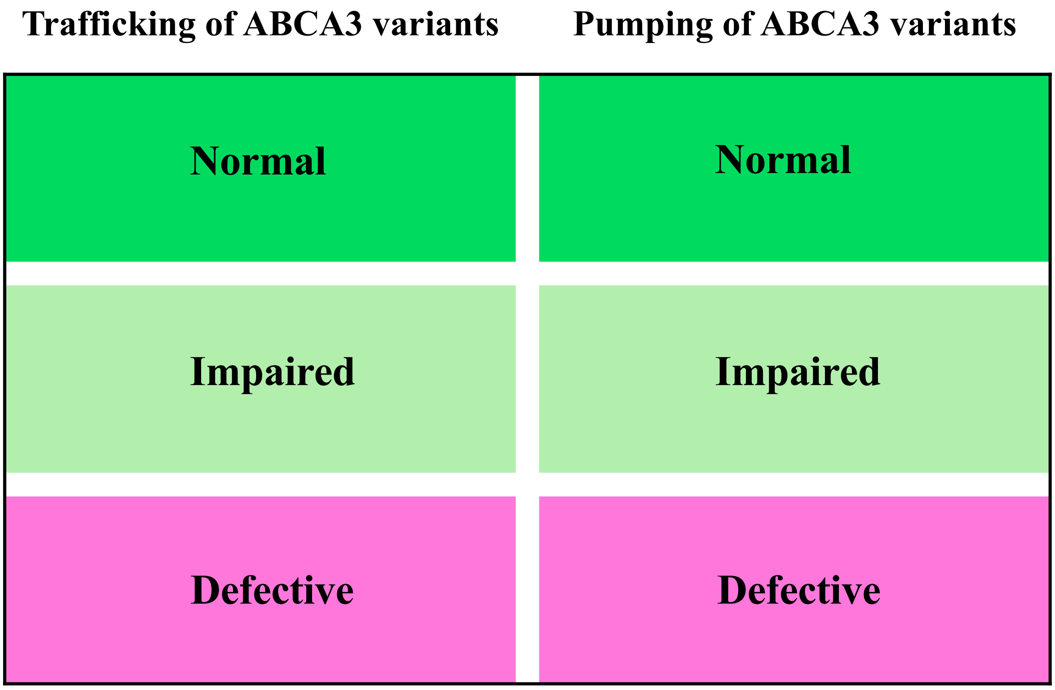

Figure S3: Categories of dysfunction of ABCA3 based on quantitative results of trafficking and pumping assays. Three groups of disease related ABCA3 variants were differentiated: normal trafficking but impaired pumping, impaired trafficking and impaired pumping, defective trafficking and defective pumping.

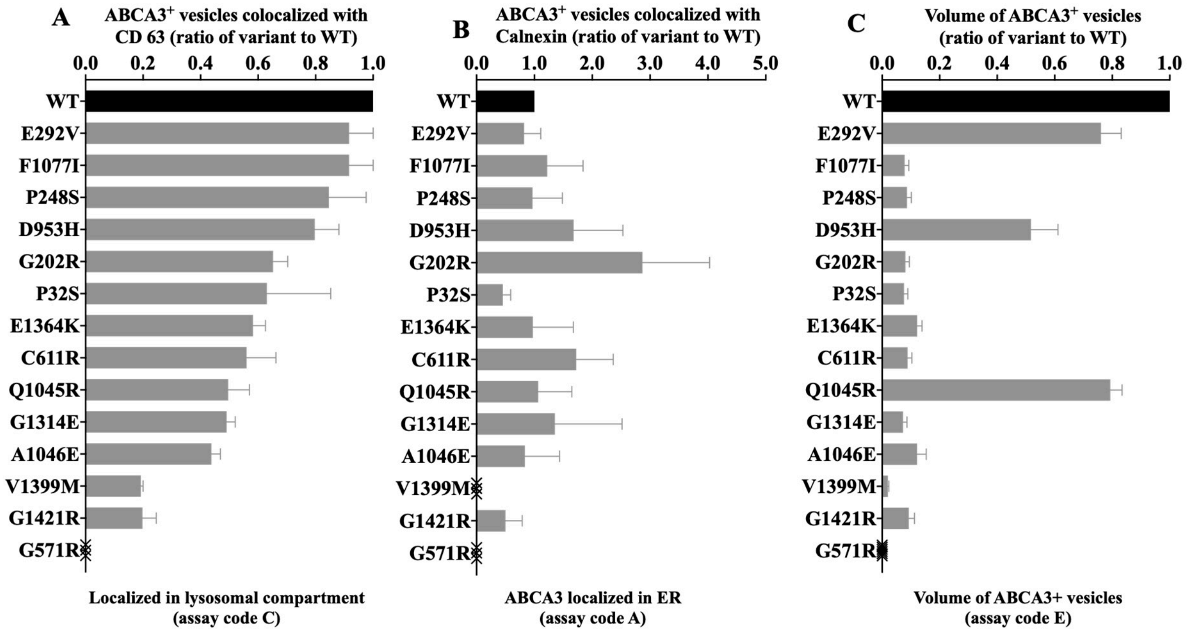

Figure S4: Quantitative characterization of novel ABCA3 variants: ABCA3+ vesicles colocalized with CD63 (A), with calnexin (B), and mean volume of ABCA3+ vesicles (C). Results were shown as Mean ± S.E.M (a - c). Cross stands for value 0.

These novel ABCA3 variants *in vitro* displayed various colocalization of ABCA3+ vesicle with lysosomal compartment marker CD63 and ER marker calnexin, with significant smaller or even undatable ABCA3+ vesicle. Based on the grouping method in this study, there were a variant with normal trafficking but impaired pumping (E292V), 12 variants with both impaired trafficking and pumping (F1077I, P248S, D953H, G202R, P32S, E1364K, C611R, Q1045R, G1314E, A1046E, V1399M, G1421R), and one variant with both defective trafficking and pumping (G571R).

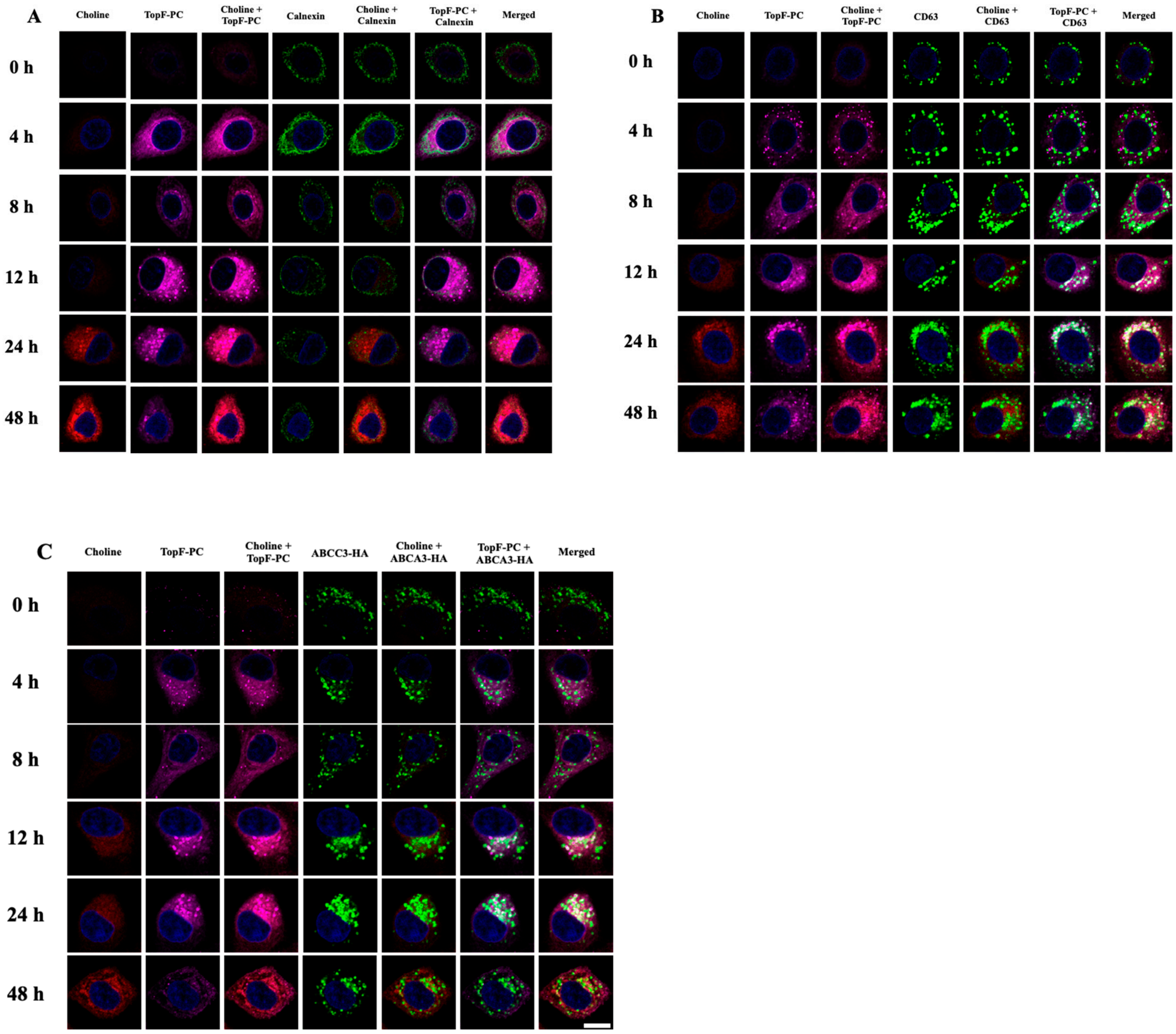

Figure S5: A549 cells stably express ABCA3-HA WT were incubated with 1:5 TopF-PC and 150 μM propargyl-choline for different time (0 h, 4 h, 8 h, 12 h, 24 h, 48 h) and then stained for endoplasmic reticulum (ER) marker calnexin (A), lysosomal compartment marker CD63 (B) and ABCA3-HA (C). Confocal images at different time points were taken. The results were consistent with the former experiments (100 μM propargyl-choline, n = 2; 1:20 TopF-PC, n = 2; data not shown). Scale bar: 10 μm.

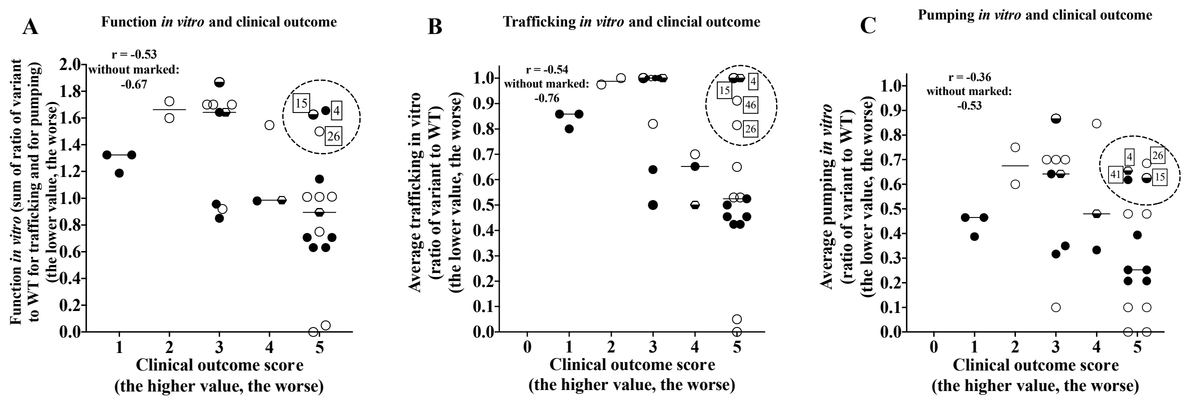

Figure S6. Correlation of clinical outcome in patients by score and sum value of function *in vitro* of ABCA3 variants (A), and average value of trafficking of ABCA3 variants (B), and average value of pumping of ABCA3 variants (C). Spearman r analysis was used. Solid circle: clinical outcomes of patients with compound heterozygous variants. Hollow circle: clinical outcomes of patients with homozygous variants. Semi-hollow circle: clinical outcomes of patients with homozygous variants and with lung transplanted. Semi-trapezoid: clinical outcomes of patients with compound heterozygous variants and with lung transplanted. The values without compatible results were marked with patients' ID (suppl. tab 5).

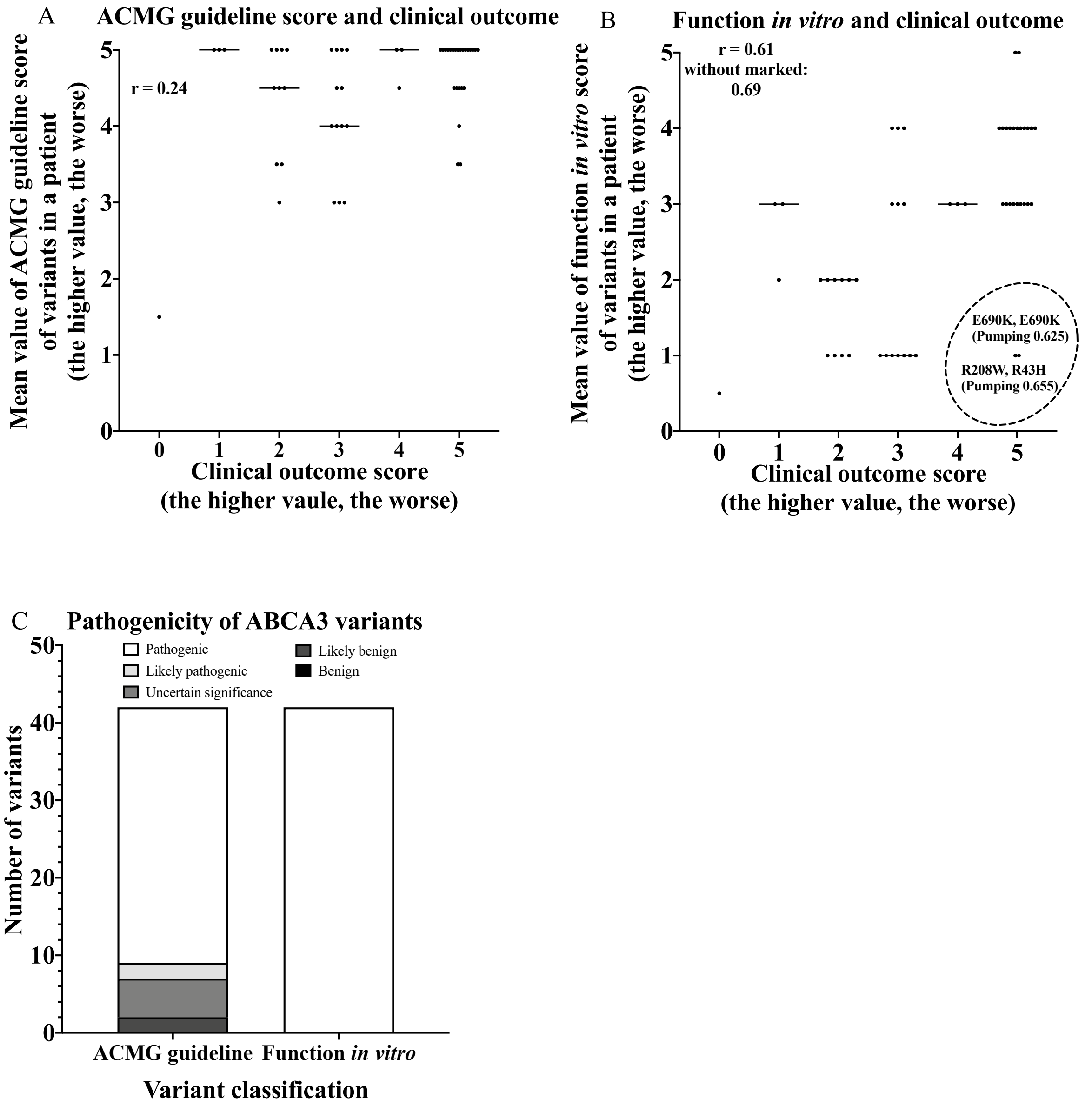

Figure S7: Correlation of clinical outcome and ABCA3 variants by ACMG guideline score (A), and ABCA3 variants by function *in vitro* score (B). Pathogenicity of ABCA3 variants in this study according to ACMG guideline score and function *in vitro*, respectively (C).

Function *in vitro*, ABCA3 variant with normal trafficking and normal pumping was scored as 0, with normal trafficking but impaired pumping was scored as 1, with normal trafficking but defective pumping was scored as 2, with impaired trafficking and impaired pumping was scored as 3, with impaired trafficking and defective pumping was scored as 4, and with defective trafficking and defective pumping as 5. For the variants unknown in allele 2 or not done (ND) *in vitro* experiments, assumed scoring was conducted as follows. If the clinical outcome score was 0, and ACMG guideline score < 3, then the variant function in vitro was assumed as (0). If the clinical outcome score was 1 or 2, and ACMG guideline score ≥ 3, then the variant function in vitro was assumed as (1). If the clinical outcome score was 3 or 4, and ACMG guideline score ≥ 3, then the variant function in vitro was assumed as (3). If the clinical outcome score was 5, and ACMG guideline score ≥ 3, then the variant function *in vitro* was assumed as (5).

Reference

1. Hu, J.Y., et al., *Functional characterization of four ATP-binding cassette transporter A3 gene (ABCA3) variants.* Hum Mutat, 2020. **41**(7): p. 1298-1307.
2. Schindlbeck, U., et al., *ABCA3 missense mutations causing surfactant dysfunction disorders have distinct cellular phenotypes.* Human mutation, 2018. **39**(6): p. 841-850.
3. Matsumura, Y., et al., *Characterization and classification of ATP-binding cassette transporter ABCA3 mutants in fatal surfactant deficiency.* J Biol Chem, 2006. **281**(45): p. 34503-14.
4. Wittmann, T., et al., *Tools to explore ABCA3 mutations causing interstitial lung disease.* Pediatr Pulmonol, 2016. **51**(12): p. 1284-1294.
5. Matsumura, Y., N. Ban, and N. Inagaki, *Aberrant catalytic cycle and impaired lipid transport into intracellular vesicles in ABCA3 mutants associated with nonfatal pediatric interstitial lung disease.* Am J Physiol Lung Cell Mol Physiol, 2008. **295**(4): p. L698-707.
6. Beers, M.F., et al., *Disruption of N-linked glycosylation promotes proteasomal degradation of the human ATP-binding cassette transporter ABCA3.* Am J Physiol Lung Cell Mol Physiol, 2013. **305**(12): p. L970-80.
7. Park, S.K., et al., *Identification and characterization of a novel ABCA3 mutation.* Physiol Genomics, 2010. **40**(2): p. 94-9.
8. Campo, I., et al., *A large kindred of pulmonary fibrosis associated with a novel ABCA3 gene variant.* Respiratory Research, 2014. **15**(1): p. 43.
9. Flamein, F., et al., *Molecular and cellular characteristics of ABCA3 mutations associated with diffuse parenchymal lung diseases in children.* Hum Mol Genet, 2012. **21**(4): p. 765-75.
10. Wambach, J.A., et al., *Functional Characterization of ATP-Binding Cassette Transporter A3 Mutations from Infants with Respiratory Distress Syndrome.* Am J Respir Cell Mol Biol, 2016. **55**(5): p. 716-721.
11. Wambach, J.A., et al., *Functional Genomics of ABCA3 Variants.* Am J Respir Cell Mol Biol, 2020. **63**(4): p. 436-443.
12. Weichert, N., et al., *Some ABCA3 mutations elevate ER stress and initiate apoptosis of lung epithelial cells.* Respir Res, 2011. **12**(1): p. 4.
13. Kinting, S., et al., *Functional rescue of misfolding ABCA3 mutations by small molecular correctors.* Hum Mol Genet, 2018. **27**(6): p. 943-953.
14. Kinting, S., et al., *Potentiation of ABCA3 lipid transport function by ivacaftor and genistein.* J Cell Mol Med, 2019. **23**(8): p. 5225-5234.
15. Beers, M.F. and S. Mulugeta, *The biology of the ABCA3 lipid transporter in lung health and disease.* Cell Tissue Res, 2017. **367**(3): p. 481-493.
16. Li, Y., et al., *Metabolic labelling of choline phospholipids probes ABCA3 transport in lamellar bodies.* Biochim Biophys Acta Mol Cell Biol Lipids, 2019. **1864**(12): p. 158516.
17. Hoppner, S., et al., *Quantification of volume and lipid filling of intracellular vesicles carrying the ABCA3 transporter.* Biochim Biophys Acta Mol Cell Res, 2017. **1864**(12): p. 2330-2335.
18. Patterson, C.E., et al., *Fatty acid synthesis in the fetal lung: relationship to surfactant lipids.* Biochim Biophys Acta, 1986. **878**(1): p. 110-26.
19. Nijssen, J., et al., *Phospholipid-protein interactions in rat lung lamellar bodies.* Biochimica et Biophysica Acta (BBA)-Lipids and Lipid Metabolism, 1987. **917**(1): p. 140-147.
